# Deciphering the molecular basis of abiotic stress response in cucumber (*Cucumis sativus* L.) using RNA-Seq meta-analysis, systems biology, and machine learning approaches

**DOI:** 10.1038/s41598-023-40189-3

**Published:** 2023-08-09

**Authors:** Zahra Zinati, Leyla Nazari

**Affiliations:** 1 Department of Agroecology, College of Agriculture and Natural Resources of Darab, Shiraz University, Shiraz, Iran; 2 Crop and Horticultural Science Research Department, Fars Agricultural and Natural Resources Research and Education Center, Agricultural Research, Education and Extension Organization (AREEO), Shiraz, Iran

**Keywords:** Computational biology and bioinformatics, Molecular biology

## Abstract

Abiotic stress in cucumber (*Cucumis sativus* L.) may trigger distinct transcriptome responses, resulting in significant yield loss. More insight into the molecular underpinnings of the stress response can be gained by combining RNA-Seq meta-analysis with systems biology and machine learning. This can help pinpoint possible targets for engineering abiotic tolerance by revealing functional modules and key genes essential for the stress response. Therefore, to investigate the regulatory mechanism and key genes, a combination of these approaches was utilized in cucumber subjected to various abiotic stresses. Three significant abiotic stress-related modules were identified by gene co-expression network analysis (WGCNA). Three hub genes (*RPL18*, *δ-COP*, and *EXLA2*), ten transcription factors (TFs), one transcription regulator, and 12 protein kinases (PKs) were introduced as key genes. The results suggest that the identified PKs probably govern the coordination of cellular responses to abiotic stress in cucumber. Moreover, the C2H2 TF family may play a significant role in cucumber response to abiotic stress. Several C2H2 TF target stress-related genes were identified through co-expression and promoter analyses. Evaluation of the key identified genes using Random Forest, with an area under the curve of ROC (AUC) of 0.974 and an accuracy rate of 88.5%, demonstrates their prominent contributions in the cucumber response to abiotic stresses. These findings provide novel insights into the regulatory mechanism underlying abiotic stress response in cucumber and pave the way for cucumber genetic engineering toward improving tolerance ability under abiotic stress.

## Introduction

Cucumber (*Cucumis sativus* L.) is one of the most popular vegetables, consumed globally, and economically significant. In agriculture, plants face adverse environmental conditions such as low and high temperatures, drought, and salinity during their life cycles, negatively impacting growth and crop productivity^1^. Due to the expansion of root networks in shallow soil and the need for high oxygen, plants exhibit weak tolerance under water stress, which causes economic damage^2,3^. During waterlogging stress and the resulting oxygen deficiency, the root system is adversely affected, leading to a deficiency in nutrient uptake^4^. The waterlogging stress in cucumbers could lead to subsequent physiological changes, such as a decrease in the photosynthesis rate^5^. Also, the cucumber yield is highly affected by salinity stress at different growth stages depending on salinity severity, salt concentration, and time exposure. Cucumber seeds, produced under salinity, show chlorosis signs along with drops in various aerial parts and root network growth criteria^6^. In addition to salinity and waterlogging, temperature stress could harmfully affect the cucumber’s growth and development from physiological and molecular aspects. Cold and high-temperature stress cause dramatic molecular response modulation. Although cucumber is known as a thermophilic vegetable, high temperatures above 35 °C cause oxidative damage to seedlings^7^, interfere with protein synthesis, and cause denaturation of proteins^8^. These could lead to a decline in the quality and yield of fruit. However, few investigations have been conducted for improving heat-tolerant cucumber cultivars through breeding and genetic engineering programs^8^. There are few studies on the response of cucumber to abiotic stresses through RNA-Seq. However, the study conducted by Wang et al.^9^ involved transcriptomic analysis of two cucumber genotypes, one drought-tolerant and the other drought-sensitive, using RNA-Seq under both normal and drought stress conditions. The results indicated that 1008 transcripts were differentially expressed under normal conditions, whereas 2265 transcripts were differentially expressed under drought stress. The study identified genes that were either up-regulated or down-regulated in response to drought stress, including those involved in sucrose and abscisic acid (ABA) signaling pathways. Li et al.^10^ conducted a comprehensive bioinformatics analysis of the cucumber genome to identify 35 GRAS genes and their role in responding to various abiotic stresses using RNA-Seq and qRT-PCR. The study showed that GRAS genes are involved in responding to abiotic stresses, such as salt, drought, and cold stress, and play an essential role in regulating plant growth and development.

Regarding the crosstalk between the molecular mechanisms through which these environmental stresses are perceived and responded to, a comprehensive study covering all these abiotic stresses could clarify the undiscovered details as well as the underlying commonality between them.

It is possible to improve the inferential power to find out pertinent biology by using combined data. Meta-analysis provides research information from different studies by collecting extensive data from individual investigations^11^. In the case of plant abiotic stress adaptations, meta-analysis uncovers the crucial responsive genes linked to the stress condition^12^. Considering the adaptation of cucumber to abiotic stresses, unraveling how different abiotic stresses can trigger plant responses could hopefully be addressed by integrated transcriptomic studies of abiotic stresses through meta-analysis. Although relatively important research was performed on some other plant species, there is no information regarding the cucumber, as an important vegetable that is adversely affected by abiotic stresses. Identification of deferentially expressed genes (DEGs) responsive to multi-abiotic stress may allow further dissection of the molecular basis of each stress and their interactions^13^. Investigating abiotic stress-responsive DEGs in Arabidopsis resulted in the identification of root and shoot-responsive genes under cold, drought, heat, and salt stresses. Similarly, Smita et al.^14^ explored the specific genes involved in multiple abiotic stresses based on a meta-analysis. In addition, several gene networks, including jasmonate, salicylic acid, and ABA signaling networks and ubiquinone pathways were recognized through co-expression analysis in their study^14^. Weighted Gene Co-expression Network Analysis (WGCNA) is regarded as an effective approach for organizing thousands of genes into manageable modules. It has been extensively used to better apprehend the molecular mechanisms of various plant species^15–17^.

In this study, we focused on those environmental stresses that mainly affect cucumber productivity. To date, no research has been conducted on gene expression meta-analysis of cucumber plants under environmental stresses, despite the fact that it would be incredibly informative. To this end, we conducted a meta-analysis on RNA-Seq gene expression data related to different experiments under cold, heat, salt, drought, and waterlogging stresses to identify the DEGs. WGCNA, along with Module Membership (MM) & Gene Significance (GS) analysis was performed to discover significant abiotic stress-related modules. Gene Ontology (GO) and Kyoto Encyclopedia of Genes and Genomes (KEGG) enrichment analyses were done to figure out the biological meaning behind the genes in the significant modules. As well, based on the module analysis, the hub genes that may be mainly attributed to the studied stresses were recognized. Moreover, given the significance of regulatory proteins, including transcription regulators (TRs), transcription factors (TFs), and protein kinases (PKs) in response to abiotic stress, efforts were made to identify these proteins in modules related to abiotic stress. Finally, validation of the efficiency of the key identified genes was evaluated using a machine learning algorithm, random forest.

## Materials and methods

The bioinformatics analysis, including data quality control, mapping, expression quantification, and normalization was done using Galaxy platform (https://usegalaxy.eu/)^18^. The batch effect, meta-Analysis of expression datasets, module identification, and cross-validation were performed using ComBat-seq, MetaDE, WGCNA, and randomForest packages in RStudio (Version 1.4.1103) as described under each subheading.

### Data collection, quality control, and mapping

The raw expression data (RNA-Seq) for a total of 96 samples across nine abiotic stress (chilling, salt, high temperature, low temperature, and waterlogging) studies in cucumber was retrieved from SRA (https://www.ncbi.nlm.nih.gov/sra) at the National Center for Biotechnology Information (NCBI). The included studies, along with their corresponding information, are listed in Table 1 and include PRJNA438923 (21 samples composed of 16 stressed and 5 control), PRJNA701131 (12 samples composed of 6 stressed and 6 control), PRJNA634519 (9 samples composed of 6 stressed and 3 control), PRJNA477930 (6 samples composed of 3 stressed and 3 control), PRJNA511946 (6 samples composed of 3 stressed and 3 control), PRJNA563457 (6 samples composed of 3 stressed and 3 control), PRJNA678740 (12 samples composed of 6 stressed and 6 control), PRJNA799460 (6 samples composed of 3 stressed and 3 control), and PRJNA844418 (18 samples composed of 9 stressed and 9 control). RNA-seq data quality was checked using FastQC v.0.11.4^19^. According to FastQC reports, the quality of the raw sequencing data submitted to SRA was sufficient for further analysis; therefore there was no need to preprocess and trim the raw expression data.

**Table 1:** Transcriptomic raw data related to abiotic stress studies of cucumber used for the current meta-analysis.

Accession	Reference	Website	Abiotic stress	Project samples	Sub-study	Treatment samples	Treatment sample information	Control samples	Control sample information
PRJNA438923		https://www.ncbi.nlm.nih.gov/bioproject/?term=PRJNA438923	Chilling	21	B*	6	Exposed to an air temperature of 6 °C for 2 h	5	Untreated
					C	5	Exposed to an air temperature of 6 °C for 6 h	5	Untreated
					D	5	Exposed to an air temperature of 6 °C for 12 h	5	Untreated
PRJNA701131	Fu et al. 2021^75^	https://www.ncbi.nlm.nih.gov/bioproject/?term=PRJNA701131	Low temperature	12	L	3	Grafted cucumber at low temperature	3	Grafted cucumber at normal temperature
					M	3	Own-root cucumber at low temperature	3	Own-root cucumber at normal temperature
PRJNA634519	Chen et al. 2020^76^	https://www.ncbi.nlm.nih.gov/bioproject/?term=PRJNA634519	High temperature	9	H	3	Heat treatment at 42 C for 3 h	3	Untreated
					I	3	Heat treatment at 42 C for 6 h	3	Untreated
PRJNA477930	Zhu et al. 2019a^77^	https://www.ncbi.nlm.nih.gov/bioproject/?term=PRJNA477930	Salt	6	K	3	Salt stressed	3	Control
PRJNA511946	Zhu et al. 2019b^78^	https://www.ncbi.nlm.nih.gov/bioproject/?term=PRJNA511946	Salt	6	A	3	Salt-stressed cucumber roots	3	Control cucumber roots
PRJNA563457		https://www.ncbi.nlm.nih.gov/bioproject/?term=PRJNA563457	Salt	6	E	3	T	3	CK
PRJNA678740	Kęska et al. 2021^79^	https://www.ncbi.nlm.nih.gov/bioproject/?term=PRJNA678740	Waterlogging	12	F	3	DH2 1xH	3	DH2 Ctrl
					G	3	DH4 1xH	3	DH4 Ctr
PRJNA799460		https://www.ncbi.nlm.nih.gov/bioproject/?term=PRJNA799460	Waterlogging	6	J	3	WL	3	CK
PRJNA844418		https://www.ncbi.nlm.nih.gov/bioproject/?term=PRJNA844418	Waterlogging	18	N	3	Hypocotyl basic tissue of Zaoer-N(ME) cucumber under waterlogging stress for 48 h	3	Hypocotyl basal tissue of Zaoer-N(CME) cucumber control treatment for 48 h
					O	3	Hypocotyl vascular bundle of Zaoer-N(VB) cucumber under waterlogging stress for 48 h	3	Hypocotyl vascular bundles treated with Zaoer-N(CVB) cucumber control for 48 h
					P	3	Hypocotyl epidermis of Zaoer-N(SK) cucumber under waterlogging stress for 48 h	3	Hypocotyl epidermis of Zaoer-N (CSK) cucumber control treated for 48 h

*To reduce heterogeneity and obtain more accurate and reliable results, the studies were divided into sub-studies. Each uppercase alphabet indicates a sub-study.

The *Cucumis sativus* reference genome was obtained from the Ensembl Plants database in FASTA and GFF formats. Quality-checked reads were mapped onto the (*Cucumis sativus* L.) reference genome sequence via Hisat2^20^. Based on a study by Kim et al.^20^, HISAT2 was chosen over other alignment tools such as OLego, STAR, and TopHat2 for its advantages in terms of memory efficiency, processing speed, and alignment accuracy.

### Expression quantification via featureCounts

In this study, featureCounts^21^ was chosen for read counting due to its advantages over HTSeq, as reported in previous studies. Specifically, featureCounts is faster^21^, more accurate in detecting and counting multi-mapping reads, especially those that overlap with multiple exons^21^, more memory-efficient^21^, and demonstrated higher sensitivity and accuracy in detecting differentially expressed genes in RNA sequencing data from cancer samples compared to HTSeq^22^. Moreover, BAM files from Hisat2 were used as input-aligned files. Based on the information available from studies on the SRA website, the nature of the RNA-Seq data used in our analysis is non-stranded. Therefore, unstranded read counting was chosen, and “exon” was specified as the feature type so that only rows that have the matched exon in the provided GTF annotation file were included for read counting.

### Normalization and batch effect

To make an accurate comparison between samples, normalization is an essential step. DESeq2^23^ was used to normalize the count data obtained from FeatureCounts. DESeq2’s median ratios have been reported to perform well for comparisons of gene count between samples and differential expression analysis^24^. Sequencing depth and RNA composition factors are taken into count in this normalization method. To be more specific, counts are divided by the size specified by the sample determined by the median ratio of gene counts pertinent to the geometric mean per gene^25^.

Adjustment of batch effects for RNA-Seq count data was carried out using ComBat-seq^26^ (Supplementary File, Sheet S1). ComBat-seq adopts a negative binomial regression model to protect the integer nature of count data in RNA-Seq studies and make the batch-adjusted data compatible with standard differential expression software packages that need integer counts. Superior statistical power and false positive control are achieved by ComBat-seq in differential expression in comparison with using other methods to adjust the data^26^.

### Meta-analysis of expression dataset

Gene expression analysis was conducted using the MetaDE package^27^. MetaDE package brings about 12 major meta-analysis methods for differential expression analysis. Rank Product^28^ was used for detecting DEGs involved in abiotic stress response. The adjusted *p*-values (FDR < 0.0001) were considered significant and were used for further analysis. MetaDE has been specifically designed for the meta-analysis of differential expression (DE) data. The aim of this package is to combine the p-values from multiple studies to identify genes that are differentially expressed between two or more experimental conditions. However, one of the limitations of p-value combination methods is the presence of heterogeneity in the data, which may arise due to differences in effect sizes or variability between studies. To address this issue, the studies can be divided into subgroups, or sub-studies, which can help to identify potential biases or sources of heterogeneity in the data, thereby improving the accuracy and reliability of the meta-analysis. For instance, in the case of study PRJNA438923, we divided the study into three sub-studies denoted as B, C, and D, respectively. These sub-studies were designed to examine the effects of exposing three samples to an air temperature of 6 °C for 2, 6, and 12 h, respectively, as compared to three control samples. By dividing the study into sub-studies, we can reduce heterogeneity and obtain more accurate and reliable results. It should be noted that when conducting meta-analyses, heterogeneity is a crucial factor that needs to be taken into consideration to avoid inaccurate or misleading conclusions.

### Module identification

To create a correlation network for DEGs and clusters (modules), WGCNA R package^29^ was used. The module contains highly correlated genes. These modules are utilized to screen core genes to respond to abiotic stress in cucumbers. In other words, with the help of a Pearson correlation, a similarity matrix was created ([Sij =|0.5 + 0.5 ∗ cor (xi, xj)|]). In this formula, Sij represents the similarity score between two items (i) and (j), while cor(xi, xj) represents the Pearson correlation coefficient between the expression profiles (xi) and (xj) of items (i) and (j). The formula for Sij combines the correlation coefficient with a constant value of 0.5 to produce a similarity score that ranges from 0.5 to 1.5, with higher values indicating greater similarity between the expression profiles of the two genes. The absolute value function ensures that the similarity score is always positive. Using a β equal to 15 as a soft-thresholding power, it was turned into an adjacency matrix ([Aij = (|0.5 + 0.5 ∗ cor (xi, xj)|)β]). The formula Aij = (|0.5 + 0.5*cor(xi, xj)|)β combines the similarity score with the soft-thresholding parameter β to produce an adjacency score that reflects the strength of the connection between two items. In other words, the parameter β is a soft-thresholding parameter that is used to transform the similarity scores into adjacency scores. To delete outliers and samples, the hclust function was employed with clustering method “average”. To identify the modules, a dynamic tree cut method was utilized based on dissTOM hierarchical clustering and other parameters including minModuleSize of 30 and deepSplit of 2. The modules with dissimilarity coefficients of 0.1 (cutHeight = 0.1) were aggregated to achieve the final co-expression modules. In addition, the first principal component of the expression data of each module is determined as a module eigengene for that module using the moduleEigengenes function. Then, the moduleTraitCor and moduleTraitPvalue functions were used to calculate the correlation coefficients of sample stress status with module eigengene values and corresponding *p*-values, respectively. The labeled heatmap plot of correlations between sample stress status and co-expression modules, with each cell containing a correlation coefficient and *p*-value, was generated based on the correlation coefficients using the labeledHeatmap function of the WGCNA package.

### Identification of significant modules and Hub genes

Module membership (MM) was specified as an association of genes and a given module, which described the reliability of a gene that belongs to a module, and gene significance (GS) was assumed as the correlation between genes with stress status. Studies have shown that when GS and MM are extensively correlated, there is an intense association between the key elements in the modules and trait^30^. Therefore, these significant modules can be utilized to develop the network and determine the hub genes.

Cytoscape v3.9.1 was used to visualize the co-expression network in the module of interest. The file including nodes (genes) and weight (the co-expression strength between two nodes), obtained from WGCNA analysis, was used as Cytoscape input *(*Supplementary file; Sheet S2). The edges signifying the correlations in each significant module were filtered by a condition of the weight value being greater than 0.6. The key genes were found using Cyto-Hubba, which is a Cytoscape Plugin for hub object analysis in the network. Algorithms for finding hub genes were an ongoing research topic in computer science. In a co-expression network, the Maximal Clique Centrality (MCC) algorithm was suggested to be the most efficient method to find hub nodes^31^. Using MCC, a topology-based scoring method, the nodes are assigned scores. For each significant module, the gene with the top MCC value was considered a hub gene (Supplementary file; Sheet S3).

### Gene ontology and pathway annotation of significant modules

To characterize the functions and processes of the genes within each module, as well as to identify the pathways in which they are involved, we performed gene ontology and pathway annotation using DAVID (Database for Annotation, Visualization and Integrated Discovery) (https://david.ncifcrf.gov/)^32^ with the given parameters. The pathway enrichment analyses were performed using the KEGG database^33^, which is integrated into the DAVID bioinformatics resources.

### Identification of DEGs encoding transcription factors, transcription regulators, and protein kinase

To determine TRs, TFs, and PKs and then classify individual TRs, TFs and PKs into different gene families, the protein sequences corresponding to the differential genes were retrieved from the Ensembl Plants (http://plants.ensembl.org)^34^ and blasted against the database of iTAK^35^ with an E-value cutoff of 10^−5^.

### Promoter motif analysis

To extract the 1000 base pairs (bps) upstream flanking region of genes corresponding to the significant module, Ensembl Plants (http://plants.ensembl.org) was used. Then, MAST (Motif Alignment & Search Tool) in The MEME suite (https://meme-suite.org/meme/index.html) was used to search sequences for matches to a set of motifs and sort the sequences by the best combined match to all motifs^36^.

### Validation of key genes

We performed tenfold cross-validation of identified key genes including three hub genes, 10 TFs, 1 TR, and 12 PKs to classify samples in stress and control conditions. To this end, Random Forest as a common machine learning algorithm^37^ with 500 trees was implemented using randomForest package^38^ in R Studio version 4.1.2. Construction of the model was based on 90% of the dataset for training set and the resting 10% for testing the model.

## Results

### Meta-analysis of expression dataset

To identify common DEGs in cucumbers exposed to various abiotic stresses, RNA-Seq data were retrieved from independent experiments, consisting of 55 samples of plants exposed to chilling, low temperature, high temperature, salt, and waterlogging and 44 samples of control plants. From 23,629 genes, 3487 genes were detected as DEGs Including 1902 up and 1585 down (Supplementary File, Sheet 4).

### WCGNA and modules significance calculation

**Figure 1:**
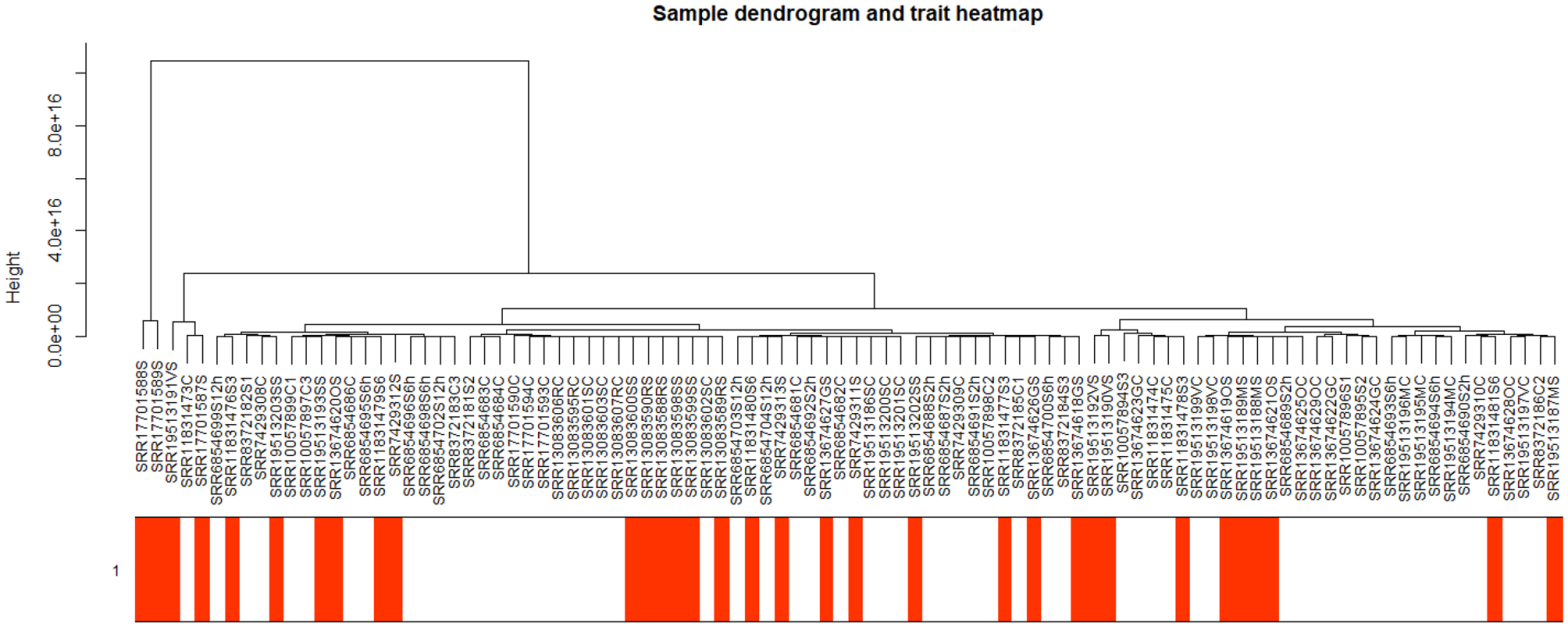
A sample dendrogram and trait heatmap were generated to reveal the clustering patterns of samples based on expression data. The clustering dendrogram of the 96 samples (41 controls and 55 abiotic stress samples) was constructed using the average clustering method. The accompanying heatmap illustrates the traits corresponding to each sample, with the white color indicating the control condition and the red color indicating the abiotic stress condition.

**Figure 2:**
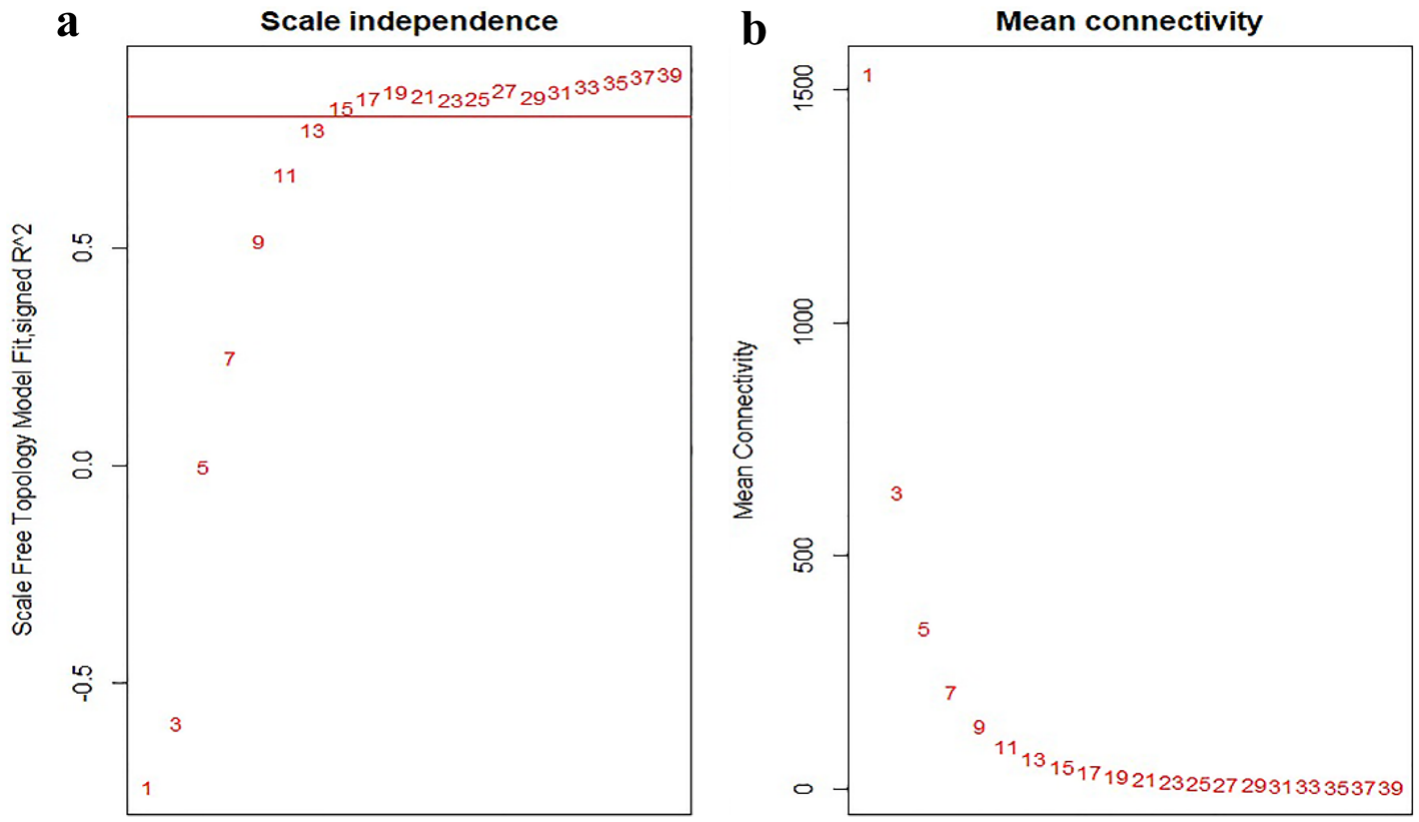
The scale independence and mean connectivity for different soft-thresholding powers (β) were analyzed using the WGCNA. (**a**) and (**b**) show the scale independence and mean connectivity of different β values, respectively, with the red line indicating the selected cut-off value. To ensure high-scale independence (approximately 0.8) and low mean connectivity (approximately 0), the β value was set to 15.

**Figure 3:**
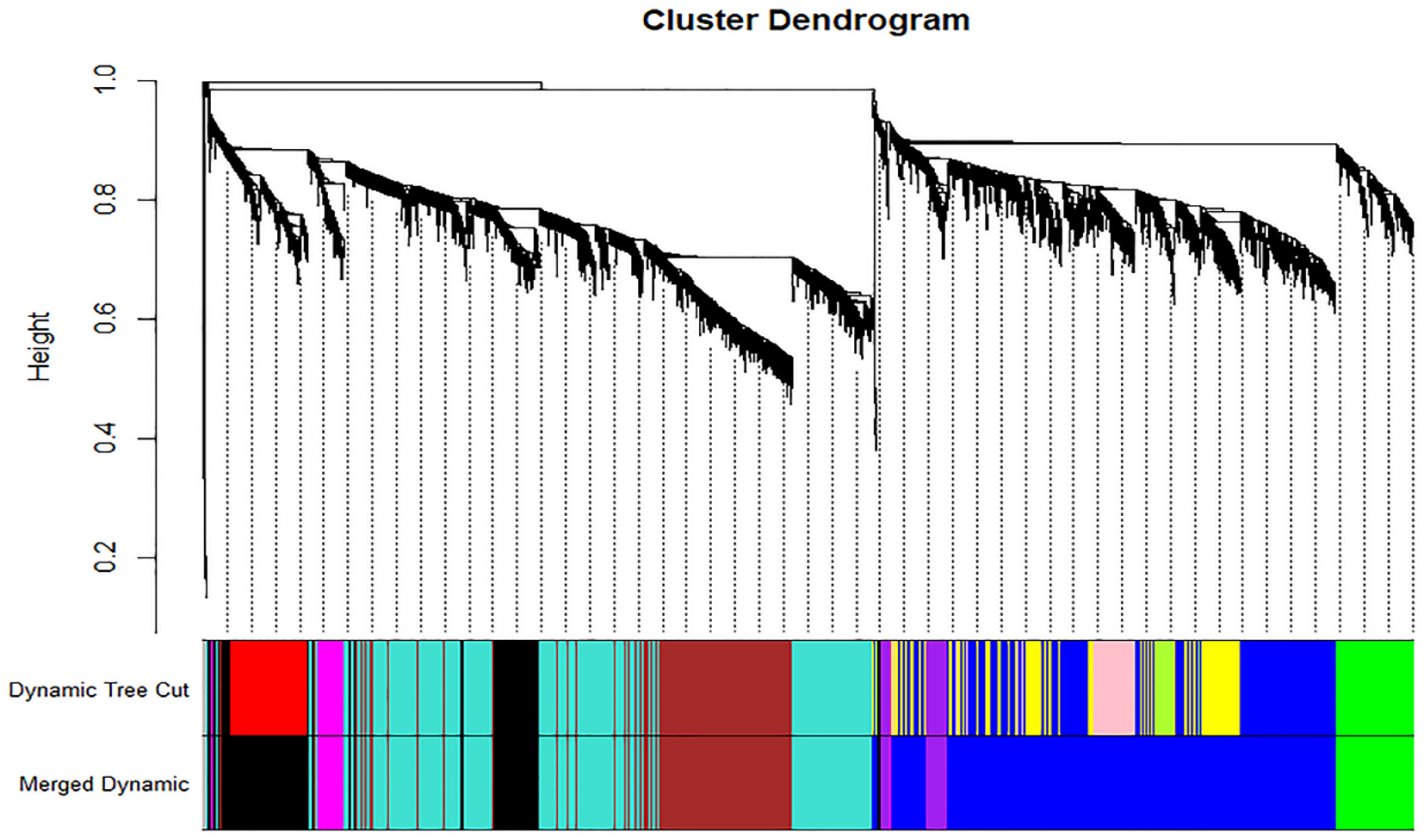
By utilizing the WGCNA package, a dendrogram was constructed to cluster genes based on their topological overlap. Each module was assigned a unique color, resulting in the creation of 12 co-expression modules that were displayed in different colors. The merging of modules with dissimilarity coefficients of 0.1 led to the creation of 8 modules, namely blue, purple, brown, turquoise, magenta, black, green, and grey.

**Figure 4:**
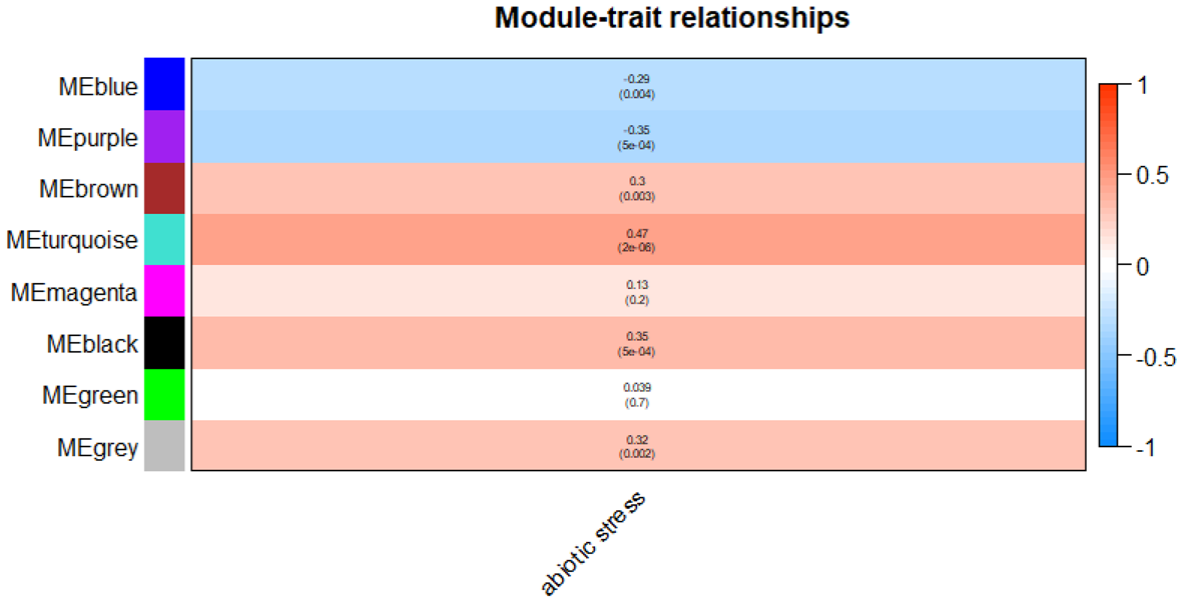
Module-trait relationship heatmap using WGCNA package. Each cell contains the corresponding correlation score and *p*-value. Red indicates a positive correlation while blue indicates a negative correlation. The table is color-coded by correlation according to the color legend.

**Figure 5:**
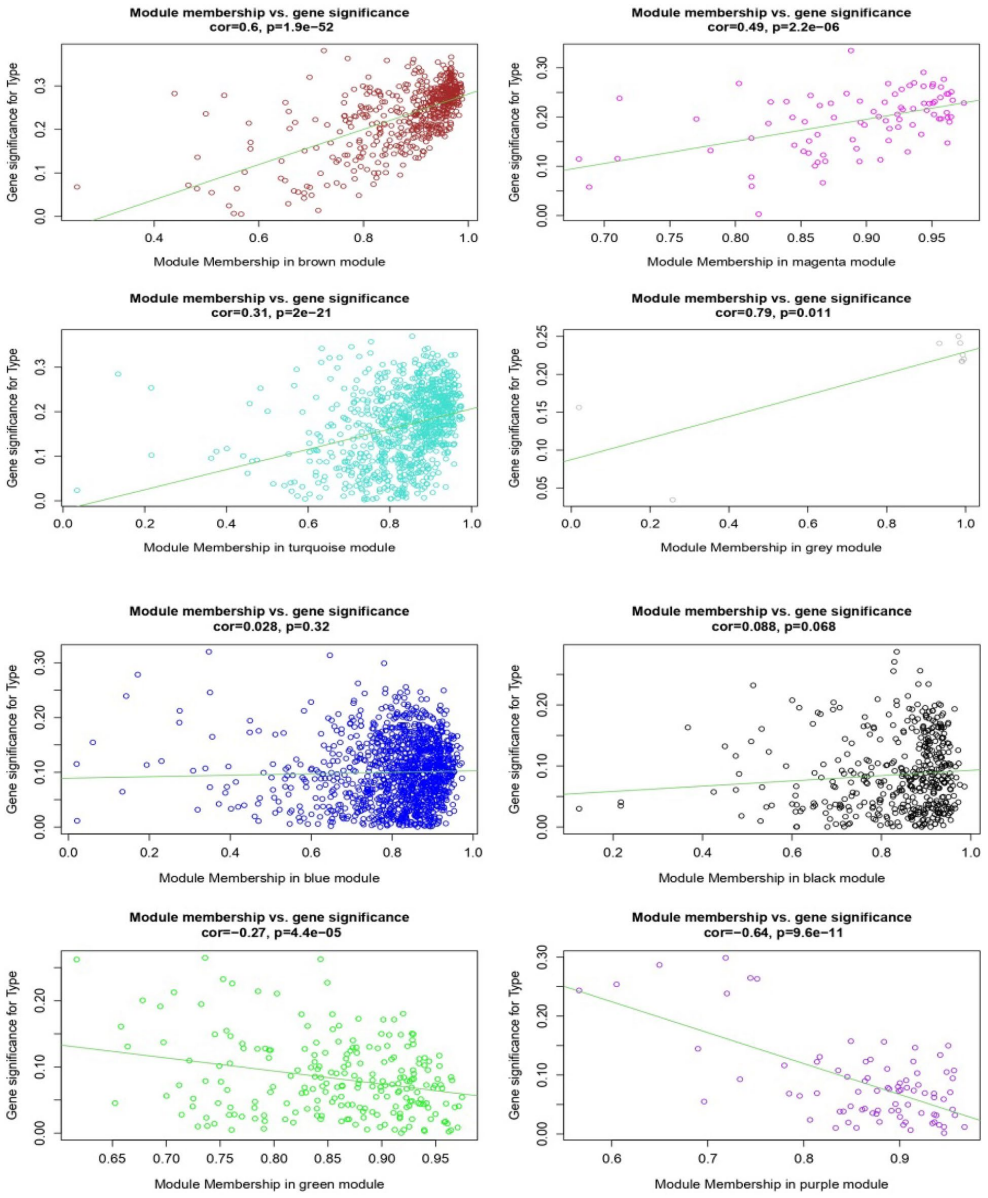
The relationship between module membership (MM) and gene significance (GS) was further statistically investigated in eight modules using GS & MM analysis via the WGCNA package. MM refers to the strength of the association between a gene and a particular module, and GS is defined as the correlation between gene expression and stress status. GS & MM positively correlated indicates strong association between key elements in modules and the trait. The grey, brown, and magenta modules had high correlation coefficients (0.79, 0.6 and 0.49, respectively) and low p-values (0.011, 1.9e–52 and 2.2e–06, respectively), suggesting their potential role in the cucumber’s response to stress.

The adjusted normalized dataset of DEGs containing 96 samples (41 controls and 55 stresses) was used for weighted gene correlation network analysis. Genes were classified into different modules using the WGCNA package. Figure 1 illustrates the dendrogram and abiotic stress status heatmap. To warrant high scale independence (about 0.8) and low mean connectivity (about 0), the value of β was set equal to 15 (Fig. 2). The value of dissimilarity of the modules was equal to 0.1 and a total of eight modules were obtained (Fig. 3). The module trait relationship is illustrated in Fig. 4. The membership module and the significance of genes are highly correlated (0.79, 0.6, and 0.49) with low *p*-value (0.011, 1.9e-52, and 2.2e-06) in the grey, brown, and magenta modules respectively (Fig. 5). Therefore, these modules can be used to identify the hub genes pertinent to the abiotic stress response.

### TFs, TR, and PKs in significant modules

The brown module contained 10 TFs belonging to five families, the largest of which was C2H2 with five members, and a transcription regulator GNAT. The only grey module TF belonged to C2C2-GATA. Also, regarding PKs, there were three and seven members of CAMK and RLK-Pelle proteins, respectively in the brown module, and two members of RLK-Pelle proteins in magenta. The information on TFs, TR, and PKs is shown in Table 2.

**Table 2:** Transcription factors, transcription regulators, and protein kinases in significant modules.

Module	Cucumber gene ID	Genes	Family	Regulatory genes	Regulation
Brown	LOC101212699	AT2G37060|NF-YB8	NF-YB	Transcription factors	Up
	LOC101214947	AT2G37120	S1Fa-like	Transcription factors	Up
	LOC101209434	AT2G41040|T3K9.19	C2H2	Transcription factors	Up
	LOC101216401	AT3G07650|COL9	C2C2-CO-like	Transcription factors	Down
	LOC101204380	AT3G44750|HDA3	C2H2	Transcription factors	Up
	LOC101220200	AT3G50700|IDD2	C2H2	Transcription factors	Up
	LOC101219700	AT4G31420|F3L17.9	C2H2	Transcription factors	Up
	LOC101223180	AT4G37670|NAGS2	GNAT	Transcriptional regulators	Up
	LOC101204140	AT5G03740|HD2C	C2H2	Transcription factors	Up
	LOC101212433	AT5G54680|ILR3	bHLH	Transcription factors	Down
	LOC101203531	AT1G01140|CIPK9	CAMK_CAMKL-CHK1	Protein kinases group CAMK	Up
	LOC101203246	AT1G07570|APK1A	RLK-Pelle_RLCK-VIIa-2	Protein kinases group RLK-Pelle	Down
	LOC101209540	AT1G10940|SNRK2.4	CAMK_OST1L	Protein kinases group CAMK	Up
	LOC101215813	AT1G26150|PERK10	RLK-Pelle_PERK-1	Protein kinases group RLK-Pelle	Up
	LOC101223136	AT2G47060|PTI1-4	RLK-Pelle_RLCK-VIII	Protein kinases group RLK-Pelle	Up
	LOC101220050	AT3G09830	RLK-Pelle_RLCK-VIIa-2	Protein kinases group RLK-Pelle	Up
	LOC101212993	AT3G15890	RLK-Pelle_RLCK-XV	Protein kinases group RLK-Pelle	Up
	LOC101222748	AT3G24550|PERK1	RLK-Pelle_PERK-1	Protein kinases group RLK-Pelle	Up
	LOC101208203	AT3G50530|CRK	CAMK_CDPK	Protein kinases group CAMK	Up
	LOC101206762	AT4G23160|CRK8	RLK-Pelle_DLSV	Protein kinases group RLK-Pelle	Up
Grey	LOC101218275	AT4G26150|CGA1	C2C2-GATA	Transcription factors	Down
Magenta	LOC101203099	AT4G22730	RLK-Pelle_LRR-IV	Protein kinases group RLK-Pelle	Up
	LOC101219093	AT5G41260|BSK8	RLK-Pelle_RLCK-XII-1	Protein kinases group RLK-Pelle	Up

### Co-expression network construction and hub genes identification

We obtained three hub genes associated with the abiotic stress response. The identified hub genes were LOC105436293 (ribosomal protein L18; *RPL18*) in the brown module, LOC101222934 (coatomer subunit delta; *δ-COP*) in the magenta module, and LOC101207054 (expansin-like A2; *EXLA2*) gene in the grey module (Supplementary file; Sheet S3). The expression levels of the hub genes increased in response to abiotic stress (Supplementary file; Sheet S4). The figure depicting the co-expression network analysis for the key identified genes is available in supplementary file, Sheet S5.

### Gene ontology and pathway annotation of significant modules

Most notable was the upregulation of most of the genes in each module. Translation was the most significant biological process in the brown module. In addition, some GO terms related to response to stress were enriched in this module such as response to cadmium ion, response to inorganic substance, response to cold, response to endoplasmic reticulum stress, and response to heat. Furthermore, structural constituent of ribosome was the most significant GO term of molecular function. In terms of cellular components, cytosol and ribosome were enriched (Supplementary File, Sheet S6).

In the magenta module, the most significant GO terms in biological processes were intracellular protein transport, response to cadmium ion, and ubiquitin-dependent protein catabolic process. Furthermore, structural molecule activity, endopeptidase activity, and proteasome-activating ATPase activity were the most significant GO terms of molecular function. Regarding the cellular component, cytosol was enriched (Supplementary File, Sheet S7).

In the grey module, in terms of biological process, the most significant GO terms were plant-type cell wall loosening and unidimensional cell growth. Furthermore, the plant-type cell wall was the most significant cellular component term (Supplementary File, Sheet S8).

KEGG pathway enrichment analysis was performed on significant modules. Brown module was significantly related to nine pathways, including ribosome (ath03010), biosynthesis of amino acids (ath01230), citrate cycle (TCA cycle) (ath00020), carbon metabolism (ath01200), glycolysis/gluconeogenesis (ath00010), carbon fixation in photosynthetic organisms (ath00710), 2-Oxocarboxylic acid metabolism (ath01210), oxidative phosphorylation (ath00190), and protein processing in endoplasmic reticulum (ath04141). Also, magenta module was significantly associated with proteasome (ath03050) and glutathione metabolism (ath00480) (*p*-value ≤ 0.05) (Supplementary File, Sheets S6, S7 and S8).

### Cross-validation of key genes

**Figure 6:**
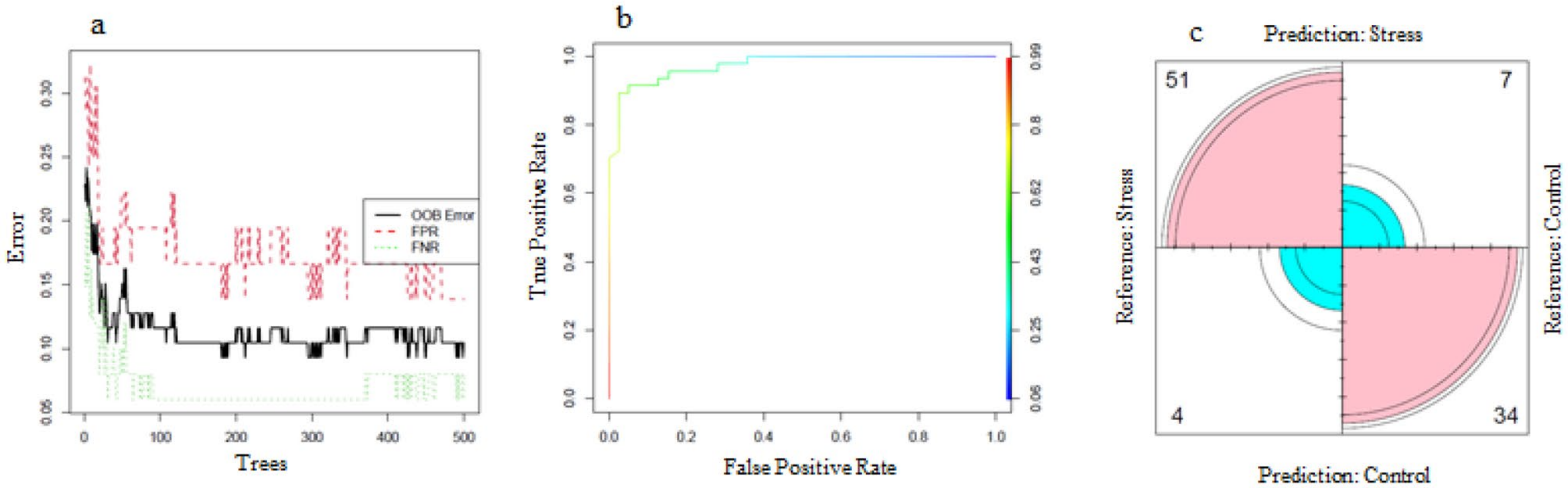
Graph of validation of out-of-bag error (OOB) rate, false positive rate (FPR), and false negative rate versus number of trees (**a**), receiver operating characteristic (ROC) (**b**), and confusion matrix (**c**) of random forest algorithm for efficacy of hub genes in classification of abiotic stress and control conditions in cucumber.

We used the identified 26 genes (3 hub genes, 10 TFs, 1 TR, and 12 PKs) as minimal genes that can accurately discriminate between samples in control and stress conditions. These genes may be considered the key genes involved in plant response under abiotic stress. A plot showing the error rate vs. the number of the tree is presented in Fig. 6. The performance of the Random Forest algorithm using these 26 genes as input was evaluated through out-of-bag error (OOB), False Positive Rate (FPR), False Negative Rate (Fig. 6a), Receiver operating characteristic (ROC) (Fig. 6b), and confusion matrix (Fig. 6c). An OOB error was estimated to be 10.47% which is made for the cases which were not used while building the tree. The area under the curve of ROC (AUC) was obtained equal to 0.974 and the accuracy rate of the model was 88.5%.

## Discussion

Higher changes in temperatures and highly changed rainfall patterns brought on by extreme weather conditions and climate change led to drought and/or flooding, which in turn affected the salinity of soil^39^. Therefore, there is a growing interest in improving crop plants genetically more tolerant to stress.

Plants usually face several stresses at the same time, so the development of cultivars tolerant to several stresses via new biotechnological methods is necessary. The development of tolerant cultivars to a wide range of abiotic stress called for the identification of common responsive hub genes to various abiotic stresses. Considering the availability of transcriptomic datasets of cucumber in response to various abiotic stresses, meta-analysis can be a suitable approach to identify common genes that respond to a wide range of different abiotic stresses. On the other hand, unwanted variation (batch effects) between datasets caused by technical variation and experimental conditions may reduce the profit of merging data for the increase of statistical power^26^ and have an unfavorable impact on downstream biological analysis. Accordingly, adjustment of batch effect for RNA-Seq count data was performed using ComBat-seq. We obtained 3,487 DEGs comparing abiotic stress and normal conditions by MetaDE package. The WGCNA method gave us an extensive characterization of the gene expression alteration for stress’s functional interpretation and resulted in a novel perspective of the molecular mechanisms of abiotic stress. There are not many reports on using WGCNA to determine co-expression networks related to abiotic stress response in cucumbers^6^. To achieve this gap, we performed a WGCNA approach by obtaining module-trait correlations based on the adjusted normalized dataset congaing 96 samples (41 controls and 55 stresses), and 3447 genes. Significantly, eight definite gene modules were identified based on the expression data of differential genes from different studies, among them, three modules (grey, brown, and magenta) were highly related to the abiotic stress response according to MM & GS analysis and further analyzed based on biological processes and biological pathways. Also, using the MCC algorithm, hub genes were identified in important modules. Examining the ontology of important modules related to abiotic stress made it clear that a series of biological processes are enriched in these modules. All processes, biological pathways, and hub genes of modules related to abiotic stress are discussed.

Regulatory proteins may function in stress signal transduction by influencing the expression of downstream functional genes. These regulatory proteins include transcription factors, protein kinases, protein phosphatases, and proteins associated with inorganic phosphate (Pi) turnover^40^. In light of the important role of regulatory proteins in abiotic stress response, efforts were made to identify these proteins in significant modules resulting in 10 TFs, one TR, and 12 PKs. CAMK and RLK-Pelle were two PK families identified in the significant modules.

Receptor-like kinases perceive environmental changes in the extracellular space and transmit the signal into the cell. To this end, second messengers and reactive oxygen species are used, which interfere with phosphorylating transcription factors, abscisic acid signaling, and other unknown signaling proteins^41^. One of the PKs identified was *SnRK2*. An analysis of the expression of *SnRK* gene family under abiotic stress in cucumbers was carried out by Luo and coworkers^42^. They gave an explicit result indicating that *CsSnRKs* can participate in the development, growth, and stress response of cucumbers. In consistent to our findings, the abundance of the majority of *CsSnRK2* genes transcripts caused by abiotic stress like methyl jasmonate (MeJA), draught, and abscisic acid (ABA) treatments indicates that *CsSnRK2* genes can have a role to play in cucumber abiotic stress responses^42^. In addition, the subfamily of *SnRK2* plays a major role in regulating gene expression by activating the basic region leucine zipper (bZIP) transcription factor^43^.

C2H2 zinc finger proteins function as key regulators of abiotic stress responses in plants^40^. Several C2H2-type zinc finger proteins function as transcriptional activators in plant growth and abiotic stress^44^. According to a review by^40^, the same C2H2 zinc finger protein gene might be induced by many abiotic stresses simultaneously. Moreover, diverse C2H2 zinc finger proteins enhance the resistance of plants to stress via similar protein kinase signaling pathways^40^. There are reports that C2H2 zinc finger proteins can directly target downstream ion balance-related genes and enhance salt resistance^45^. They can also target key genes that take part in biosynthesizing osmotic adjustment substances for the enhancement of osmotic stress resistance^46^. In addition, they can target antioxidant genes related to reactive oxygen species (ROS) scavenging in abiotic stress conditions^47^, C-repeat/DRE-binding factor genes (CBFs) to enhance resistance to cold^48^, and target downstream genes taking part in hormone signal transduction^49^.

Due to the widespread presence of the PKs family and C2H2 TF members in the brown module, it seems that after receiving an abiotic signal, PKs such as *CIPK9*, *APK1A*, *SNRK2.4*, *PERK10*, *PTI1-4*, *PERK1*, *CRK*, and *CRK8* might phosphorylate the downstream C2H2 TFs, which consecutively inhibit or activate stress-related genes containing binding sequences for C2H2 TFs.

To mine the potential candidate targets of C2H2 TFs, the 1000 base pairs upstream flanking region of co-expressed genes in the brown module were explored whether they contained binding sequences for C2H2 TFs such as A(G/C)T repeat sequences^50^, A[AG/CT]CNAC^40^, TGCTANNATTG element^51^, TACAAT motifs^52^. The results showed that seven genes co-expressed with C2H2 TFs contained one TGCTANNATTG element in their promoters. These genes included AT1G47420 (succinate dehydrogenase 5; *SDH5*), AT1G59900 (pyruvate dehydrogenase complex E1 alpha subunit; *E1 ALPHA*), AT1G60710 (NAD(P)-linked oxidoreductase superfamily protein; *ATB2*), AT3G01280 (voltage dependent anion channel 1; *VDAC1*), AT3G04790 (Ribose 5-phosphate isomerase, type A protein; *EMB3119*), AT5G28060 (Ribosomal protein S24e family protein; *RPS24B*), and AT4G02450 (HSP20-like chaperones superfamily protein; *P23-1*). Additionally, target genes of C2H2 TFs are upregulated in a similar pattern to C2H2 TFs in the abiotic stress studies used in this research including chilling, low temperature, high temperature, salt, and waterlogging. A large body of evidence indicates that these target genes participate widely in the response to various environmental stresses^53–60^.

The coordinated expression of these genes with C2H2 TFs, along with the presence of binding sequences for C2H2 TFs in their promoters, suggest that they are targets of C2H2 TFs. Co-expression between these identified C2H2 zinc finger proteins and several target downstream stress-related genes in the brown module may imply the irreplaceable role of this family in abiotic stress response in cucumber. Further investigations are needed to scrutinize the functions of PKs and TFs and their associated signaling pathways in cucumber abiotic stress responses.

The brown module is related to the biological process of translation, and most of the proteins encoded by the genes of this module are located in the cytosol and ribosome. Also, the ribosome pathway and amino acid biosynthesis were the most significant pathways in the brown module. It was interesting that the brown module also contained GO-terms of the abiotic stress responses. In addition, a set of genes had potential roles in the response to numerous stresses. For instance, AT4G24280 (*cpHsc70-1*) in response to cadmium ion, response to cold, and response to heat; AT3G09630 (Ribosomal protein L4/L1 family) in response to cadmium ion, and response to inorganic substance; AT1G09780 (*iPGAM1*), AT5G20720 (*CPN20*), AT2G33800 (*EMB3113*) in response to cadmium ion, and response to cold; AT2G47470 (*UNE5*) in response to cadmium ion, and response to endoplasmic reticulum stress; AT4G23100 (*GSH1*), AT3G04120 (*GAPC1*), AT1G79930 (*HSP91*) in response to cadmium ion, and response to heat; AT3G59080 (Eukaryotic aspartyl protease family protein) in response to cold, and response to inorganic substance; AT4G24920 (secE/sec61-gamma protein transport protein) response to endoplasmic reticulum stress, and response to inorganic substance; AT5G12380 (*ANNAT8*), AT1G79440 (*ALDH5F1*), AT3G53110 (*LOS4*) in response to cold, and response to heat. One unexpected GO-term was a response to cadmium ion that drew our attention. An explanation for this could be sharing the same signal transduction pathway with other stress-related TFs by Cd-responsive TFs^61^. Therefore, it can be activated by other abiotic stresses like dehydration, cold, SA, and H_2_O_2_. This can also imply that the plant may coincidentally experience adversities cadmium toxicity when under chilling, low temperature, high temperature, salt, and waterlogging stresses. In concert with this finding, in a study conducted by^13^ to explore gene expression patterns in abiotic stress conditions in Arabidopsis, “response to cadmium ion (GO:0,046,686)” was one of the GO terms enriched in salt-specific gene modules^13^. The hub gene in this module is ribosomal protein L18 (*RPL18*). Studies have shown that in response to environmental stresses, particularly phytohormone and cold treatments, the expression of the 34 genes responsible for coding rice ribosomal protein large subunits, including RPL18a and RPL18p, undergoes significant changes indicating their differential response to stress^40^. Additionally, knockdown experiments of 60S ribosomal protein L14-2 have demonstrated important and potential roles for this protein in enhancing the tolerance of plants to drought and salt stress^62^.

The magenta module is related to the biological processes of protein transport and catabolism, and most of the proteins coded by this module were located in the cytosol. The magenta module was related to the proteasome pathway. Biological processes of protein transport and catabolism are among the first processes that are affected by stress. The ubiquitin–proteasome system can regulate the proteins’ function which has a role in the generation of the cellular changes needed to react to the changing environment and lower the negative effect of stress^63,64^. When exposed to external stimuli, ubiquitination is inhibited or promoted, which results in a higher degradation or stabilization of transporters, enzymes, ion channels, transcription regulators (like co-activators, transcription factors, and repressors), and signaling proteins (receptors and kinases). These alterations in protein abundance can enhance or prevent cellular responses^65^. According to the MCC algorithm, *δ-COP* was the hub gene in this module. *δ-COP* is a constituent protein of the coatomer complex, which plays a role in regulating the trafficking of proteins between the Golgi apparatus and the endoplasmic reticulum within cells^66^. Studies have indicated the involvement of protein trafficking and the endomembrane system in the response of plants to abiotic stress^10,67^. Loss of function of the beta-COP subunit, one of the components of the coatomer complex, has been shown to affect Golgi structure, plant growth, and salt stress tolerance in Arabidopsis^68^. Therefore, it is possible that the coatomer complex, including the delta subunit, may play a role in the response of cucumber to abiotic stress, although the specific function of the delta subunit is yet to be fully understood.

The gray module is related to the biological process of cell wall modification, and most of the proteins coded by this module were placed in the cell wall. According to the MCC algorithm, the expansin-like A2 (*EXLA2*) gene was defined as the hub gene in this module. This finding is consistent with Abuqamar et al.^69^ who reported that knockout of the Arabidopsis expansion gene *AtExLA2* resulted in longer roots compared to wild species with normal growth conditions and at the same time made roots more susceptible to salt stress^69^. Expansin genes encode for plant cell wall-related proteins which control plant cell division and contribute to the growth regulation of plant tissues^70^. *CsEXPB-04* and *CsEXPA-11* which are expansin genes are confirmed to play a role in abiotic stress responses in cucumber based on expression analysis^71^. The loosening of cell wall polysaccharides by loosening enzymes like expansins implies its importance under drought, osmotic, or salt stress to continue the growth of stressed cells and organs^41^.

In this research, we implemented Random Forest model to evaluate the efficacy of hub genes in distinguishing the abiotic stress and control condition based on gene expression values. Accuracy rate was 88.5% along with 0.974 AUC indicating the efficiency of our model. Machine learning algorithms have been frequently applied to classify binary or multi-class instances. Different evaluation metrics have been employed to validate the classifier performances. The commonly used methods are Cross-Validation, Confusion Matrix, and Receiver Optimizer Characteristics (ROC)^72^. Validation has previously been implemented on gene expression values of hub genes^73,74^.

## Conclusions

The development of cultivars that are tolerant to numerous abiotic stresses may be facilitated by the identification of common key responsive genes to various abiotic stresses, which is of particular importance in breeding programs. In this investigation, a meta-analysis covering genome-wide transcriptional profiling of cucumber in response to various abiotic stresses was carried out. WGCNA was utilized in an effort to narrow down the 3487 DEGs identified through meta-analysis and in turn characterize key and common genes involved in various abiotic stresses. WGCNA along with MM & GS analysis resulted in the identification of three significant modules related to abiotic stress, including grey, brown, and magenta modules. So, these modules were screened for hub genes and regulatory proteins. Hub module genes included *RPL18* in the brown module, *δ-COP* in the magenta module, and *EXLA2* gene in the grey module, according to MCC algorithm. Additionally, ten TFs, one TR, and twelve PKs were identified. Regarding the widespread presence of C2H2 TFs such as *T3K9.19*, *HDA3*, *IDD2*, *F3L17.9*, and *HD2C* as well as the PKs such as *CIPK9*, *APK1A*, *SNRK2*.*4*, *PERK10*, *PTI1-4*, *PERK1*, *CRK*, and *CRK8* in the brown module, co-expression analysis, and promoter analysis it appears that after receiving an abiotic signal, PKs might phosphorylate the downstream C2H2 TFs, which consecutively inhibit or activate stress-related genes containing binding sequences for C2H2 TFs including *SDH5*, *E1 ALPHA*, *ATB2*, *VDAC1*, *EMB3119*, *RPS24B*, and *P23-1*. According to GO and KEGG enrichment analysis, the most significant biological processes and pathways associated with abiotic stress-related modules in cucumber were translation, intracellular protein transport, plant-type cell wall loosening, ribosome, and proteasome pathway.

We used Random Forest to validate the efficacy of the key genes. High AUC and accuracy values of Random Forest model may demonstrate that these are key genes that have a prominent contribution in the responses of cucumber plants when exposed to various abiotic stresses. The level of expression of these genes can be compared between genotypes presenting different stress tolerance under stress conditions to identify the genes that contribute to a general tolerance to different abiotic stress conditions. Although bioinformatics analyses can be powerful in identifying genes associated with stress, experimental validation through techniques such as overexpression and knockout studies, as well as gene expression analysis using qRT-PCR, is essential to confirm the results and obtain precise details. After the validation of genes, they can be developed as biomarkers for breeding abiotic stress-tolerant cucumber varieties. This finding not only sheds light on plant stress responses and adaptations, but it also builds a foundation for breeding programs.

### Supplementary Information

Supplementary Information 1.

Supplementary Information 2.

## Data Availability

All data generated or analysed during this study are available from the corresponding authors on reasonable request.
