# Supplementary material for: Deciphering the molecular basis of abiotic stress response in cucumber (Cucumis sativus L.) using RNA-Seq meta-analysis, systems biology, and machine learning approaches
Source: Sci Rep. 2023 Aug 9;13:12942. doi: 10.1038/s41598-023-40189-3 (PMC10412635; doi:10.1038/s41598-023-40189-3)
Supplement: Supplementary file 1 — Supplementary Information 1. [file 41598_2023_40189_MOESM1_ESM.docx]

source("http://bioconductor.org/biocLite.R")

biocLite("MetaDE")

library(MetaDE)

setwd("D:/projects/Zinati/deg/RR")

getwd()

study.names<-c("A","B","C","D","E","F","G","H","I","J","K","L","M","N","O","P")

#Read data sets into R (merge and filter data)

mydata.raw<-MetaDE.Read(study.names,skip=rep(1,16),via="txt",matched=T,log=F)

#mydata.matched<-MetaDE.match(mydata.raw,"IQR")

mydata.merged<-MetaDE.merge(mydata.raw,MVperc=0)

#filtered out 30% un-expressed genes and then 30% non-informative genes#

mydata.filtered<-MetaDE.filter(mydata.merged,c(0.0,0.0))

########################################################

#Identify differentially expressed genes in each individual dataset

ind.Res1<-ind.analysis(mydata.filtered,ind.method=rep("modt",16),nperm=1000,tail="abs")

head(ind.Res1$p)

write.csv(ind.Res1$p, file = " results of individual study.csv")

###################################

MetaDE.Res<-MetaDE.rawdata(mydata.filtered,ind.method=rep("modt",16),meta.method=c("rankProd"),nperm=1000)

Up_result<-MetaDE.Res[["FDR.up"]]

Down_result<- MetaDE.Res[["FDR.down"]]

write.csv(Up_result, file = "Up_result (FDR-value).csv")

write.csv(Down_result, file = "Down_result (FDR-value).csv")

AveFC<-MetaDE.Res[["AveFC"]]

write.csv(AveFC, file = "AveFC.csv")

meta.stat.up<-MetaDE.Res[["meta.stat.up"]]

write.csv(meta.stat.up, file = "meta.stat.up.csv")

meta.stat.down<-MetaDE.Res[["meta.stat.down"]]

write.csv(meta.stat.down, file = "meta.stat.down.csv")

pval.up<-MetaDE.Res[["pval.up"]]

write.csv(pval.up, file = "pval.up.csv")

pval.down<-MetaDE.Res[["pval.down"]]

write.csv(pval.down, file = "pval.down.csv")

#To get significant genes counts

#Set working directory

setwd("D:/projects/Zinati/wgcna total genes/final batch normalized 0.0001 meta")

dir()

#Reading count table

genes<-read.table(choose.files(), header = TRUE, row.names = 1)

head(genes)

lNormexpr<-read.table(choose.files(), header = TRUE, row.names = 1)

sigGenes = match(rownames(genes), rownames(lNormexpr))

significantCounts <- lNormexpr[sigGenes,]

write.csv(significantCounts, file = "SigCounts.csv")

#Co-Expression Analysis using WGCNA

# Load the WGCNA package

library(WGCNA)

# The following setting is important, do not omit.

options(stringsAsFactors = FALSE);

enableWGCNAThreads()

#it can be RNA seq data or micro array

ExpressionData = significantCounts

# ExpressionData = ExpressionData[,-c(1:5)]

# colnames(ExpressionData) <- gsub("X.media.ad.9117284696_AD.FASTQ_Files_Bulk.", "", colnames(ExpressionData))

# colnames(ExpressionData) <- gsub(".Sorted.bam", "", colnames(ExpressionData))

transposed_ExpressionData = as.data.frame(t(ExpressionData));

#filter high NA values and zero variance genes and samples

gsg = goodSamplesGenes(transposed_ExpressionData, verbose = 3);

gsg$allOK

if (!gsg$allOK)

{

# Optionally, print the gene and sample names that were removed:

if (sum(!gsg$goodGenes)>0)

printFlush(paste("Removing genes:", paste(names(transposed_ExpressionData)[!gsg$goodGenes], collapse = ", ")));

if (sum(!gsg$goodSamples)>0)

printFlush(paste("Removing samples:", paste(rownames(transposed_ExpressionData)[!gsg$goodSamples], collapse = ", ")));

# Remove the offending genes and samples from the data:

transposed_ExpressionData = transposed_ExpressionData[gsg$goodSamples, gsg$goodGenes]

}

# Outlier detection using hierarchical clustering

sampleTree = hclust(dist(transposed_ExpressionData), method = "average");

#pdf(file = "Plots/sampleClustering.pdf", width = 12, height = 9);

par(cex = 0.6);

par(mar = c(0,4,2,0))

{plot(sampleTree, main = "Sample clustering to detect outliers", sub="", xlab="", cex.lab = 1.5,

cex.axis = 1.5, cex.main = 2)

# adjust abline to make a better cut

abline(h = 35, col = "red") }

# Remove outliers here

# Plot a line to show the cut

# Determine cluster under the line

clust = cutreeStatic(sampleTree, cutHeight = 35, minSize = 10)

table(clust)

# select which cluster samples you want to keep.

keepSamples = (clust == 0)

transposed_ExpressionData = transposed_ExpressionData[keepSamples, ]

nGenes = ncol(transposed_ExpressionData)

nSamples = nrow(transposed_ExpressionData)

# Load pheno data

traitData = read.csv("Pheno.csv", row.names = 1);

# to be in same order like transposed expression data

traitData = traitData[rownames(transposed_ExpressionData),]

collectGarbage()

# Re-cluster samples

sampleTree2 = hclust(dist(transposed_ExpressionData), method = "average")

# Convert traits to a color representation: white means low, red means high, grey means missing entry

traitColors = numbers2colors(traitData, signed = FALSE);

plotDendroAndColors(sampleTree2, traitColors,

groupLabels = names(traitData),

main = "Sample dendrogram and trait heatmap")

save(transposed_ExpressionData, traitData, file = "dataInput.RData")

#=====================================================================================

#

# Let's make network

#

#=====================================================================================

# Choose a set of soft-thresholding powers

powers = c(seq(from = 1, to=40, by=2));

# Call the network topology analysis function

sft = pickSoftThreshold(transposed_ExpressionData, powerVector = powers, verbose = 5)

# Plot the results:

par(mfrow = c(1,2));

cex1 = 0.9;

# Scale-free topology fit index as a function of the soft-thresholding power

{plot(sft$fitIndices[,1], -sign(sft$fitIndices[,3])*sft$fitIndices[,2],

xlab="Soft Threshold (power)",ylab="Scale Free Topology Model Fit,signed R^2",type="n",

main = paste("Scale independence"));

text(sft$fitIndices[,1], -sign(sft$fitIndices[,3])*sft$fitIndices[,2],

labels=powers,cex=cex1,col="red");

# this line corresponds to using an R^2 cut-off of h

abline(h=0.80,col="red")}

# Mean connectivity as a function of the soft-thresholding power

plot(sft$fitIndices[,1], sft$fitIndices[,5],

xlab="Soft Threshold (power)",ylab="Mean Connectivity", type="n",

main = paste("Mean connectivity"))

text(sft$fitIndices[,1], sft$fitIndices[,5], labels=powers, cex=cex1,col="red")

#put first value which cut the 0.9 as soft power

softPower = 15;

adjacency = adjacency(transposed_ExpressionData, power = softPower, type = "signed")

#corFnc = "bicor"

#=====================================================================================

#

# Similarity hierarchical clustering of genes

#

#=====================================================================================

# To minimize effects of noise and spurious associations,

# we transform the adjacency into Topological Overlap Matrix (Similarity)

TOM = TOMsimilarity(adjacency);

dissTOM = 1-TOM

# Call the hierarchical clustering function

geneTree = hclust(as.dist(dissTOM), method = "average");

# Plot the resulting clustering tree (dendrogram)

sizeGrWindow(12,9)

plot(geneTree, xlab="", sub="", main = "Gene clustering on TOM-based dissimilarity",

labels = FALSE, hang = 0.04);

#=====================================================================================

#

# Let's get modules

#

#=====================================================================================

# We like large modules, so we set the minimum module size relatively high:

#minimum genes in a module

minModuleSize = 30;

# Module identification using dynamic tree cut:

dynamicMods = cutreeDynamic(dendro = geneTree, distM = dissTOM,

deepSplit = 2, pamRespectsDendro = FALSE,

minClusterSize = minModuleSize);

table(dynamicMods)

# Convert numeric lables into colors

dynamicColors = labels2colors(dynamicMods)

table(dynamicColors)

# Plot the dendrogram and colors underneath

plotDendroAndColors(geneTree, dynamicColors, "Dynamic Tree Cut",

dendroLabels = FALSE, hang = 0.03,

addGuide = TRUE, guideHang = 0.05,

main = "Gene dendrogram and module colors")

#=====================================================================================

#

# Let's merge modules using eigengenes

#

#=====================================================================================

# Calculate eigengenes

MEList = moduleEigengenes(transposed_ExpressionData, colors = dynamicColors)

MEs = MEList$eigengenes

# Calculate dissimilarity of module eigengenes

MEDiss = 1 - cor(MEs);

# Cluster module eigengenes

METree = hclust(as.dist(MEDiss), method = "average");

# Plot the result

plot(METree, main = "Clustering of module eigengenes",

xlab = "", sub = "")

# Altitude to merge

MEDissThres = 0.1

# Plot the cut line into the dendrogram

abline(h=MEDissThres, col = "red")

# Call an automatic merging function

merge = mergeCloseModules(transposed_ExpressionData, dynamicColors, cutHeight = MEDissThres, verbose = 3)

# The merged module colors

mergedColors = merge$colors;

# Eigengenes of the new merged modules:

mergedMEs = merge$newMEs;

#Plot merged modules

#pdf(file = "Plots/geneDendro-3.pdf", wi = 9, he = 6)

plotDendroAndColors(geneTree, cbind(dynamicColors, mergedColors),

c("Dynamic Tree Cut", "Merged Dynamic"),

dendroLabels = FALSE, hang = 0.03,

addGuide = TRUE, guideHang = 0.05)

#dev.off()

# Rename to moduleColors

moduleColors = mergedColors

# Construct numerical labels corresponding to the colors

colorOrder = c("grey", standardColors(50));

moduleLabels = match(moduleColors, colorOrder)-1;

table(moduleLabels)

MEs = mergedMEs;

# Save module colors and labels for use in subsequent parts

save(MEs, moduleLabels, moduleColors, geneTree, file = "networkConstruction-stepByStep.RData")

############################################################################################################

# Load the WGCNA package

library(WGCNA)

# The following setting is important, do not omit.

options(stringsAsFactors = FALSE);

# Load the expression and trait data saved in the first part

lnames = load(file = "dataInput.RData");

# Load network data saved in previous step

lnames = load(file = "networkConstruction-stepByStep.RData");

# Define numbers of genes and samples

nGenes = ncol(transposed_ExpressionData);

nSamples = nrow(transposed_ExpressionData);

# Recalculate MEs with color labels

MEs0 = moduleEigengenes(transposed_ExpressionData, moduleColors)$eigengenes

MEs = orderMEs(MEs0)

moduleTraitCor = cor(MEs, traitData, use = "p");

moduleTraitPvalue = corPvalueStudent(moduleTraitCor, nSamples);

# Will display correlations and their p-values

textMatrix = paste(signif(moduleTraitCor, 2), "\n(",

signif(moduleTraitPvalue, 1), ")", sep = "");

dim(textMatrix) = dim(moduleTraitCor)

par(mar = c(6, 8.5, 3, 3));

# Display the correlation values within a heatmap plot

labeledHeatmap(Matrix = moduleTraitCor,

xLabels = "abiotic stress", #names(traitData)

yLabels = names(MEs),

ySymbols = names(MEs),

colorLabels = FALSE,

colors = blueWhiteRed(50),

textMatrix = textMatrix,

setStdMargins = FALSE,

cex.text = 0.5,

zlim = c(-1,1),

main = paste("Module-trait relationships"))

#=====================================

#Module Membership & Gene Significance

#=====================================

# Define variable weight containing the weight column of datTrait

traitData = read.csv("Pheno.csv", row.names = 1);

Type = traitData$status

modNames = substring(names(MEs), 3)

geneModuleMembership = as.data.frame(cor(transposed_ExpressionData, MEs, use = "p"));

MMPvalue = as.data.frame(corPvalueStudent(as.matrix(geneModuleMembership), nSamples));

names(geneModuleMembership) = paste("MM", modNames, sep="");

names(MMPvalue) = paste("p.MM", modNames, sep="");

geneTraitSignificance = as.data.frame(cor(transposed_ExpressionData, Type, use = "p"));

GSPvalue = as.data.frame(corPvalueStudent(as.matrix(geneTraitSignificance), nSamples));

names(geneTraitSignificance) = paste("GS.", names(Type), sep="");

names(GSPvalue) = paste("p.GS.", names(Type), sep="");

NumerModules=table(moduleColors)

mColor=unique(moduleColors)

j=dim(NumerModules)

sizeGrWindow(7,7);

pdf("MM_GS.pdf", width = 12, height = 8)

par(mfrow = c(2,2));

for(i in 1:j){

# Select module

module = mColor[i];

column = match(module, modNames);

moduleGenes = moduleColors==module;

verboseScatterplot(abs(geneModuleMembership[moduleGenes, column]),

abs(geneTraitSignificance[moduleGenes, 1]),

xlab = paste("Module Membership in", module, "module"),

ylab = "Gene significance for Type",

main = paste("Module membership vs. gene significance\n"),

abline = TRUE, abline.color = 3, abline.lty = 1,

cex.main = 1.2, cex.lab = 1.2, cex.axis = 1.2, col = module)

}

dev.off()

#Let's extract each madule gene names

ColorBasedGenes <- names(transposed_ExpressionData)[moduleColors == "salmon"]

write.csv(ColorBasedGenes, file = "salmon.csv" )

#==============================

#Save genes of Modules In files

#==============================

NumerModules = table(moduleColors)

mColor = unique(moduleColors)

j = dim(NumerModules)

for(i in 1:j){

b=names(transposed_ExpressionData)[moduleColors==mColor[i]]

c=mColor[i]

write.table(b, file =paste(c,".txt"),row.names=FALSE, na="",col.names=FALSE, sep=",")

}

## Random Forest

library(MASS)

library(randomForest)

data <- read.delim("tml.txt")

data <- data[,-1]

index <- sample(nrow(data),nrow(data)*0.9)

data_train <- data[index,]

data_test <- data[-index,]

data_train$class <- as.character(data_train$class)

data_train$class <- as.factor(data_train$class)

data_rf <- randomForest(as.factor(class)~., data = data_train, importance=TRUE, ntree=500)

data_rf

plot(data_rf, lwd=rep(2, 3))

legend("right", legend = c("OOB Error", "FPR", "FNR"), lwd=rep(2, 3), lty = c(1,2,3), col = c("black", "red", "green"))

data_rf_pred <- predict(data_rf, type = "prob")[,2]

library(ROCR)

pred <- prediction(data_rf_pred, data_train$class)

perf <- performance(pred, "tpr", "fpr")

plot(perf, colorize=TRUE)

unlist(slot(performance(pred, "auc"), "y.values"))

pcut <-0.05

data_rf_pred_test <- predict(data_rf, newdata=data_test, type = "prob")[,2]

data_rf_class_test <- (data_rf_pred_test>pcut)*1

table(data_test$class, data_rf_class_test, dnn = c("True", "Pred"))

library(caret)

lvs <- c("control", "stress")

truth <- factor(rep(lvs, times = c(41, 55)), levels = rev(lvs))

pred <- factor(c(rep(lvs, times = c(34, 7)), rep(lvs, times = c(4, 51))), levels = rev(lvs))

xtab <- table(pred, truth)

cm <- confusionMatrix(pred, truth)

cm$table

fourfoldplot(cm$table, color = c("cyan", "pink"))
